# Cloning and Characterization of a Novel *Drosophila* Stress Induced DNase

**DOI:** 10.1371/journal.pone.0103564

**Published:** 2014-08-01

**Authors:** Chang-Soo Seong, Armando Varela-Ramirez, Xiaolei Tang, Brenda Anchondo, Diego Magallanes, Renato J. Aguilera

**Affiliations:** 1 Border Biomedical Research Center and Department of Biological Sciences, The University of Texas at El Paso, El Paso, Texas, United States of America; 2 Department of Medicine, Loma Linda University, Loma Linda, California, United States of America; St. Georges University of London, United Kingdom

## Abstract

*Drosophila melanogaster* flies mount an impressive immune response to a variety of pathogens with an efficient system comprised of both humoral and cellular responses. The fat body is the main producer of the anti-microbial peptides (AMPs) with anti-pathogen activity. During bacterial infection, an array of secreted peptidases, proteases and other enzymes are involved in the dissolution of debris generated by pathogen clearance. Although pathogen destruction should result in the release a large amount of nucleic acids, the mechanisms for its removal are still not known. In this report, we present the characterization of a nuclease gene that is induced not only by bacterial infection but also by oxidative stress. Expression of the identified protein has revealed that it encodes a potent nuclease that has been named Stress Induced DNase (SID). SID belongs to a family of evolutionarily conserved cation-dependent nucleases that degrade both single and double-stranded nucleic acids. Down-regulation of *sid* expression *via* RNA interference leads to significant reduction of fly viability after bacterial infection and oxidative stress. Our results indicate that SID protects flies from the toxic effects of excess DNA/RNA released by pathogen destruction and from oxidative damage.

## Introduction

Our prior work with the lysosomal endonuclease DNase II revealed that this enzyme plays an important role in the innate response to bacterial infection in *Drosophila melanogaster* [1]. Flies deficient in DNase II were found to be highly sensitive to *Escherichia coli* and *Micrococcus luteus* bacterial infection, as the majority of infected flies died soon after infection [1]. In the first genome-wide analyses of genes affected by bacterial infection, the *dnase II* open reading frame (ORF) was found to be up-regulated (4.9 fold) by bacterial infection reaching maximal expression by 48 h [2]. In addition to the induction of *dnase II,* this survey identified another putative nuclease encoded by the CG9989 open reading frame (ORF) that was rapidly induced by 5.2 fold after 1.5 h of bacterial infection and its expression remained elevated up to 48 h post infection [2]. In a similar expression analysis, the CG9989 ORF was recently found to be up-regulated by ∼47 fold after 16 h of infection with a pathogenic version of *Pseudomonas entomophila* but not with an avirulent strain of this bacteria [3]. Interestingly, the CG9989 ORF was also found to be activated by 3.2 fold and 4.2 fold by oxidative stress after paraquat and hydrogen peroxide treatment, respectively [4]. Due to the induction of this putative nuclease by distinct biological and chemical stressors, we have named this protein Stress Induced DNase (SID).

SID exhibits strong peptide sequence identity with members of the sugar-non-specific nuclease family also known as the ββα-Me-finger nuclease family [5]–[7]. This ancient enzyme family consists of non-specific double- and single-stranded DNA/RNA nucleases found in both prokaryotes and eukaryotes [5]. The prototypical member of this family, for which the protein structure has been determined, is the *Serratia marcescens* nuclease, SmNuc [7]–[9]. In addition, many *Drosophila* and arthropod species (mosquitoes, beetles, shrimps, crabs, and others) were found to contain similar ORFs with lower sequence identities. The *sid*-like gene of the *Culex quinquefasciatu*s mosquito encodes an active enzyme named Cuqu Endo that is secreted in the salivary glands [10]. The SID*-*like nuclease of Kuruma prawn (*Penaeus japonicus*) is expressed in the hepatopancreatic organ where it appears to act as a digestive enzyme [11], [12]. In *D. melanogaster*, *sid* is primarily expressed in the larval and adult fat body while in the adult it is expressed in additional tissues (as reported in FlyAtlas; [13]). Apart from its expression in the fat body, *sid* expression was also detected in larval hemocytes *via* microarray analysis [14]. Interestingly, SID is weakly homologous (22% homology overall) to the well characterized human mitochondrial Endo-G nuclease that is involved in DNA degradation during cell death [6]. The majority of enzymatically active members of this nuclease family contain a conserved catalytic domain that is well conserved in the SID protein [5]–[7]. In this report, we provide proof that the SID protein is a *bona fide* nuclease and that it is activated by bacterial infection as well as oxidative stress.

## Materials and Methods

### Drosophila stocks

The CG9989 (*sid*) RNAi line was obtained from the Fly Stocks of National Institute of Genetics (NIG-FLY, Japan). The *W^1118^*, *y^1^v^1^ hop^Tum^/FM7c*, and *W^1118^;Df(3R)Exel6209,P{XP-U}Exel6209/TM6B,Tb^1^* were obtained from Bloomington Drosophila Stock Center at Indiana University. The *ubiquitin-GAL4* line was kindly provided by Dr. Utpal Banerjee at University of California, Los Angeles and the *UAS-dicer* line was obtained from Dr. Kyung-An Han at The University of Texas at El Paso. The *UAS-sid* line was generated by BestGene (Chino Hills, CA). Additionally, *sid* knock down and ubiquitous over expression lines were generated using standard genetic methods by crossing of flies carrying the *UAS-sid RNAi*, *UAS-sid*, *W^−^/Ubiquitin-GAL4;Df(3R)Exel6209,P{XP-U}Exel6209/UAS-sid RNAi, Ubiquitin-GAL4;UAS-sid RNAi*, *Ubiquitin-GAL4;UAS-sid* and *Ubiquitin*-*GAL4;UAS-dicer;UAS-sid RNAi*. All flies were maintained in standard corn meal/agar medium (110 g sucrose, 52 g cornmeal, 27.4 g yeast, 7 g agar, 2.4 g methyl p-hydroxybenzoate/8.5 ml ethanol, and 1.5 g propionic acid/L) at 25°C except that the temperature was increased to 29°C during septic infection. Flies were kept in humidified incubators programmed for 12 hour day/night periods.

### Cloning of the *sid* gene in expression vectors

The *sid* open reading frame was cloned into the cold-shock vector pCold (Takara Bio Inc., Otsu, Japan) that has been recommended for improved expression of potentially toxic genes. The following primers were utilized to amplify the *sid* gene from a full length cDNA clone (pOT2-CG9989; NCBI Accession #NP_651627) that was obtained from Drosophila Genomics Resource Center (Indiana University). The following primers used for cloning purposes were engineered with restriction sites (underlined) and poly-Histidine codons (features in brackets): 5″ CATATG[Nde-1]CCCGATCTGAAGTATATGTTG and 5′ CTCGAG[Xho-1]CTAATGGTGATGGTGATGATG[six Histidines] GCAAACACCAGTTAAGTGTGGAG[*sid* gene]. Using pOT2-CG9989 vector, the *sid* gene was also cloned into GST fusion expression vector pGEX-KG vector (GE Healthcare, Piscataway, NJ) using the following oligonucleotides: (1) 5′-GAATTC[Eco R1] CGATGCCCGATCTGAAGTATATGTTGAC-3′ (*sid*-GST forward) and 5′-CTCGAG[Xho I] CTAATGGTGGTGATGGTGATGATGGCAAACACCAGTTAAGTGTGGAG-3′ (*sid*-[His]_9_ reverse). Purified PCR fragments were cloned into pGEM-T Easy vector and subjected to DNA sequencing to verify the sequence as described in fly base prior to subcloning into the pCold and GST vectors. Once the sequence was verified, the fragments were excised and subcloned into the appropriate cloning sites of the expression vectors.

### Expression of the SID protein in bacteria

After verifying the sequence of the pCold-*sid* clone, the vector was transformed into BL21 (DE3) pLysS competent cells (NEB, Ipswich, MA) and a single colony was cultured in LB containing 100 µg/ml ampicillin at 37°C overnight. The following day, the 10 ml overnight culture was transferred into 1 L LB media containing 100 µg/ml ampicillin and further cultured at 37°C under shaking (250 rpm). After the culture reached an OD of 0.4–0.5 (600 nm), 1 mM of IPTG was added to induce expression and incubated at 15°C for an additional 24 h with shaking. The bacteria were collected by centrifugation, lysed in buffer containing 1 mg/ml lysozyme, 0.1 mM PMSF, and a protease inhibitor cocktail (Halt Protease Inhibitor Cocktail; Thermo Scientific Pierce, Rockford, IL). The cell lysate was then briefly sonicated and centrifuged at 10,000×g for 30 min at 4°C. Supernatants were collected as crude protein lysates. The His-tagged SID protein was first purified via a Ni-NTA column following the manufacturer’s instruction (Qiagen, Germantown, MD). Briefly, 2 ml of a 50% Ni-NTA slurry was mixed with 10 ml protein lysate. The solution was incubated at 4°C for 60 min under constant mixing at 150 rpm and then loaded onto an empty column. The column was washed three times with the washing buffer (50 mM NaH_2_PO_4_, 300 mM NaCl, 20 mM imidazole, pH 8.0) and the His-tagged SID was then eluted with the elution buffer (50 mM NaH_2_PO_4_, 300 mM NaCl, 250 mM imidazole, pH 8.0) four times (1 ml/elution). The Ni-NTA-purified protein was analyzed by SDS PAGE and western blots using an anti-Histidine antibody (0.1 µg/ml; mouse anti-Penta-His Antibody; Qiagen, Germantown, MD) and HRP conjugated goat-anti mouse IgG (0.13 µg/ml; Thermo Scientific, Rockford, IL). The fractions enriched with the SID protein were either desalted or further purified by a size exclusion column. Briefly, the Superose 12 10/300 GL column was calibrated using size exclusion column molecular weight markers (Sigma) and a standard curve generated. The Ni-NTA-purified SID was initially loaded onto the column calibrated with 10 mM Tris-HCl, pH 7.5 and run at flow rate of 0.5 ml/min. The fractions were analyzed by western blotting as described above. The fractions that contained the SID protein were concentrated approximately ten fold before their use in nuclease assays described in the following section.

Once the pGEX-sid vector was verified by sequencing and transformed as described in the previous section, the GST-SID fusion protein was induced for purification purposes. Overnight cell cultures were diluted 100 fold in fresh 500 mL LB broth media containing 50 µg/ml ampicillin and 34 µg/ml chloramphenicol and grown at 37°C to reach OD = 0.5, and protein expression was induced by adding 1 mM IPTG for 6 hour. The cultured cells were collected by centrifugation and solubilized with GST lysis buffer (50 mM NaHPO_4_; 500 mM NaCl; 10 mM imidazole). The resulting recombinant protein was purified using glutathione sepharose 4B resin kit (GE Healthcare, Piscataway, NJ). The ∼68 KDa GST-SID-(His)_9_ fusion protein was eluted with 2 ml of 10 mM glutathione in GST buffer (20 mM Tris-HCl, pH 8.0; 150 mM NaCl; 0.5 mM TCEP; 1 mM EDTA; 100 µM PMSF). The eluted protein was then dialyzed overnight at 4°C in one liter of dialysis buffer (10 mM Tris-Cl, pH 7.5, 50 mM NaCl, 1 mM MgCl_2,_ 10% glycerol pH 7.5) using SnakeSkin Dialysis Tubing (7 kDa MW cutoff; Thermo Fisher Scientific, Rockford, IL) and concentrated by the use of a Centricon 100 centrifugal filter (Millipore, Billerica, MA). In addition, the GST-SID-(His)_9_ fusion protein was cleaved with biotinylated thrombin, which was partially removed from the cleaved product by the use of streptavidin agarose (EMD Chemicals, Inc., Gibbstown, NJ). The purified recombinant GST-SID-(His)_9_ fusion protein and the thrombin cleaved protein product were subsequently used for western blotting and nuclease assays.

### SDS-PAGE and detection of SID expression

Purified proteins were separated by electrophoresis in 10% SDS-PAGE gels in duplicate. One gel was stained with R15 Coomassie blue to visualize the protein bands. The other gel was transferred to a polyvinylidene fluoride (PVDF) membrane (0.45 µm; Thermo Scientific, Rockford, IL). The membrane was blocked with 5% BSA/TBS (Tris Buffered Saline) at 4°C overnight followed by incubation with a mouse anti-Histidine antibody (see previous section) in 5% BSA/1 X TBST for 2 hours at room temperature. After washing three times with the TBST (TBS+0.1% Tween-20), the membrane was incubated with the secondary antibody in 5% skim milk in TBST for 1 hour at room temperature. The membrane was then washed twice with TBST and twice with TBS. The histidine-tagged protein was visualized using SuperSignal West Pico Chemiluminescent Substrate (Thermo Scientific). Crude protein extracts from single female flies were separated under denaturing conditions using 10% SDS-PAGE gel and transferred to PVDF membrane. The membranes were then blocked for 1 h at room temperature with 10% w/v skim milk powder in TBS and then incubated with rabbit anti-*sid* peptide antisera (1∶2000; Antagene, Sunnyvale, CA) at 4°C overnight. The blots were then washed as described above and then incubated for an additional 1.5 h with HRP conjugated goat-anti rabbit IgG (1∶3000; Pierce) antibodies diluted in the same buffer as previous step. Actin was used as a housekeeping loading control and labeled with rabbit anti-actin antisera (1∶3000; Sigma) and then, with HRP conjugated goat-anti rabbit IgG (Pierce), as secondary antibodies (as described above). The immune-complexes were visualized using SuperSignal West Pico Chemiluminescent Substrate (Thermo Scientific) and further processed as previously described [15].

### Analysis of nuclease activity

The nuclease activity of the SID fractions was evaluated using the following substrates: double stranded generic plasmid DNA, M13mp18 single stranded DNA (Cat #N4040S; NEB), and single stranded RNA (Cat #N0362S; NEB). A typical 20 µl reaction contained 10 mM Tris-HCl (pH 7.5 unless otherwise indicated), 2.5 mM cation, nucleic acid substrate, and the partially purified protein. The reactions were performed at 37°C at different time points depending on the assay. However, the nuclease activity could be typically visualized following 1–2 hour incubation. The reactions were then analyzed in 1.5% agarose gels under native or denaturing conditions. To analyze DNA samples under denaturing condition, a 1.5% agarose gel was made using a 1x alkaline running buffer (50 mM NaOH, 1 mM EDTA). The DNA/RNA was then loaded with a 1x alkaline loading buffer (50 mM NaOH, 1 mM EDTA, 5% glycerol, 0.04% bromophenol blue, 0.04% xylene cyanol) and the treated DNA/RNA was separated in alkaline running buffer. After electrophoresis, the gel was neutralized with 1 M Tris-HCl and 1.5 M NaCl, at pH 7.6 for 45 min, stained with 0.5 µg/ml ethidium bromide in TAE buffer for 30 min, de-stained by washing with ddH_2_O twice (10 min/each), and visualized under a UV trans-illuminator. In some experiments, partial denaturing conditions were used. Briefly, the DNA/RNA samples were mixed with a loading buffer (1 mM EDTA, 0.025% bromophenol blue, 5% glycerol), heated at 65°C for 5 mins, chilled on ice, loaded immediately on a 1.5% agarose gel, and run in the TAE running buffer.

The denaturing condition facilitated visualization of fragments of nicked nucleic acids. The gels were stained with ethidium bromide (0.5 µg/ml) and visualized with a UV trans-illuminator (Alpha Innotech, San Leandro, CA).

### Anti-SID antibody production and detection of SID by immunohistochemistry

Rabbit anti-SID peptide antibodies were generated against a synthetic peptide (aa 9–24; TILSLYFFVGSVQANC; see Figure S1A) that does not bear significant homology to other nuclease family members or other *Drosophila* proteins (determined by NCBI blast analysis). Peptide production, coupling to Keyhole Limpet Hemocyanin (KLH), and immunization were all performed by Antagene Inc. (Sunnyvale, CA). Fat bodies were collected by dissecting the 3^rd^ instar larvae with fine forceps under stereomicroscope. For detection of SID in fat bodies, tissues were fixed in freshly prepared 4% formaldehyde for 30 min at room temperature and permeabilized with high PBHT (20 mM NaH_2_PO_4_, 0.5 M NaCl, 1% Triton X-100, pH 7.4) for 1 hour at room temperature. Fixed fat bodies were blocked with low PBHT (20 mM NaH_2_PO_4_, 0.5 M NaCl, 0.2% Triton X-100, pH 7.4) containing 10% normal goat serum for 1 hour at room temperature and incubated with rabbit anti-SID peptide antiserum (1: 200) overnight at 4°C. After washing with low 1 X PBHT, the fat bodies where incubated for 2 hours at room temperature with Alexa Fluor 568 goat anti-rabbit IgG (1∶1,000; Invitrogen). To identify individual cell nuclei, DAPI staining (1 µg/ml in low PBHT) was also processed for 15 min at room temperature. After washing with low PBHT, the fat bodies were carefully aligned to the top of the slides and mounted in Vecta shield mounting medium (Vector Laboratories, Burlingame, CA) and stored at 4°C. All images were generated using a confocal fluorescence LSM 700 microscope, assisted with Zen 2009 software (Zeiss, NY).

### Monitoring the effect of needle wounds on fly viability

Aseptic experiments were performed to explore the influence of the puncture wound on fly viability. For this purpose, flies were injected (punctured) with clean tungsten needles or needles coated with a dead bacterial suspension. Clean needle punctures involved needles that were exposed to 95% ethanol and then washed in distilled water and dried before use. To prepare the dead bacterial suspension, live bacterial cultures were boiled to kill the cells, then centrifuged and the pellets resuspended in PBS essentially as previously detailed [16].

### Monitoring the number of bacterial cells *in vivo*

After infection, each fly was macerated in a sterile mortar, consisting of conical pestle fitted for 1.5 microcentrifuge tubes, mixed with 100 µl of bacterial growth medium. Whole macerated tissues were evenly distributed on a surface of LB agar (Life Technologies) containing 15 µg/ml tetracycline in Petri dishes (10 cm diameter) to grow *E. coli* and Nutrient agar (Life Technologies) to growth *M. luteus*. Agar plates were incubated at 37°C or 30°C overnight, for *E. coli* and *M. luteus* respectively (as recommended American Tissue Culture Collection; ATCC), and the total numbers of colonies were counted per fly/plate.

### Immune challenge and survival experiments

*Escherichia coli* DH5α-GFP and *Micrococcus luteus* were used in this study to challenge the immune system in *Drosophila* flies. A total of 25 virgin females (2 or 3 days old) were challenged by injection of the bacteria suspension (OD = 1.1; approximately 8.8×10^8^ cells/ml) with a tungsten needle. Following infection, flies were maintained at 29°C on regular fly medium. For the majority of experiments, injections were performed at least three independent times. Only female flies were used in these and the oxidative stress experiments (see following section) to avoid gender based influences [17]. Survival experiments were performed as described previously [1] and Kaplan-Meier survival graphs were generated with the Graphpad Prism 6 program.

### Paraquat-induced oxidative stress assay

Evaluation of the effects of oxidative stress was performed essentially as previously described [18]. For the assay, two- to three-day-old virgin females were placed in groups of 20 flies in 5 ml experimental vials, containing 1 ml of solid medium composed of 1.3% low melting agarose, 1% sucrose and 5 mM, 10 mM, or 15 mM paraquat (N, N′-dimethyl-4, 4′-bipyridinium dichloride; Ultra Scientific, North Kingston, RI). Vials were maintained at 25°C for a period of 4 days and live and dead flies were counted on daily basis and dead flies were removed every day. Experimental and control groups were tested at the same time. For each experimental point, at least three vials of each indicated genotype were included.

### Quantitative Real-Time PCR (qRT-PCR)

Total RNA was isolated from 15 adult female flies and 20 third instar larvae’s fat body using TRIzol according to the manufacturer’s instructions (Invitrogen, Carlsbad, CA). Briefly, 5 µg of total RNA was treated with 4 units of DNase I (Applied Biosystems/Ambion, Austin, TX) at 37°C for 30 min. One µg of DNase I-treated total RNA was used in cDNA synthesis with oligo (dT) 15 primers (Promega. Madison, WI). The cDNA synthesis was performed by Titan One Tube RT-PCR kit procedure (Roche Applied Science. Indianapolis, IN). One µl of 10-fold diluted cDNA was used as a template for qRT-PCR. Real-time PCR was performed on iCycler iQ Real-Time PCR Detection System (Bio-Rad, Hercules, CA) using iQ SYBR Green Supermix (Bio-Rad, Hercules, CA). The oligonucleotide primers used for RpS15Aa (housekeeping gene, CG2033) and *sid* were as follows: RpS15Aa forward: 5′-TGGACCACGAGGAGGCTAGG-3′, RpS15Aa reverse: 5′-GTTGGTTGCATGGTCGGTGA-3′, *sid* forward: 5′-TCAATGGACGCTTTATGGAA-3′, and *sid* reverse: 5′-TTAATTGATCCGACTGCCAA-3′. The cycling conditions consisted of 1 cycle of 2 min at 95°C followed by 45 cycles of 10 sec at 95°C, 15 sec at 61°C and 20 sec at 72°C. Optimized qRT-PCR reactions were performed using more than 95% high PCR efficiency with a correlation coefficient between 0.95 and 1.0, and slope range between −3.1 to −3.3 for both Rps15Aa (CG2033) and *sid* primers. Results were analyzed as previously described [1], [19].

### Statistical analysis

Each data point represents three independent experiments. Results are shown as averages with their corresponding standard deviations to show experimental variability. Two-tailed paired Student’s *t*-tests were performed to find the statistical significance among two experimental samples. To determine whether comparisons of two groups had statistical significance, a value of *P*<0.05 was considered significant.

## Results

### SID is a member of the ββα-Me-finger nuclease family

**Figure 1:**
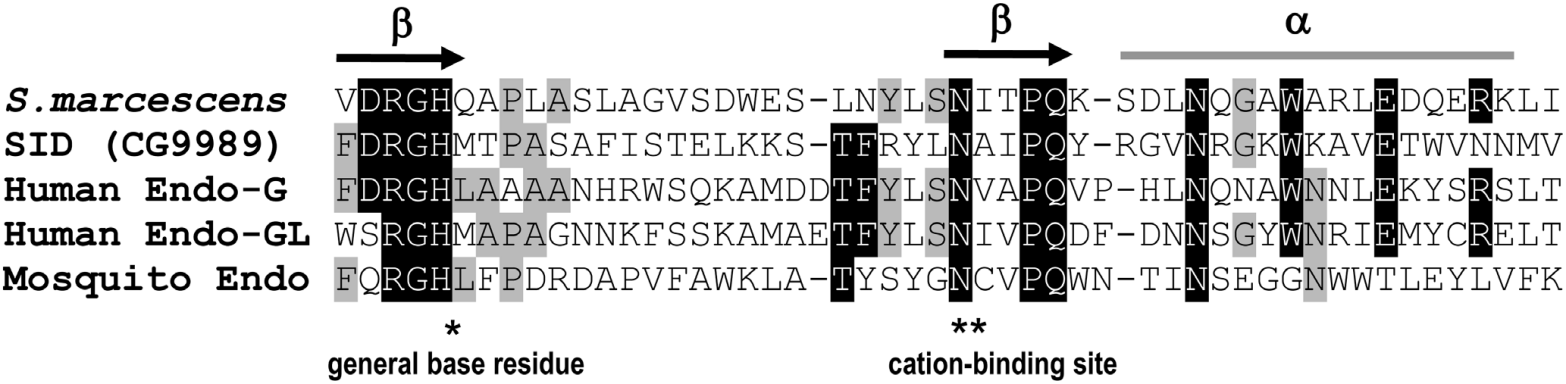
Amino acid sequence alignment of conserved elements among active sites of different nonspecific nucleases. Sequence alignment of the region containing the catalytic-residues of the SmNuc (*S.mercescens*), SID (*Drosophila*), two human mitochondrial endonucleases (Endo-G and Endo-GL), and the mosquito CuQu Endo nuclease. The compared nucleases have the conserved RGH motif called the NUC domain that is present in all active family members. The Histidine (*) within the RGH is the general based residue required for catalytic activity while the conserved Asparagine (**) is required for cation binding. Arrows on top of the aligned sequences indicate the two beta sheets and the grey line indicates the position of the alpha helix.

Members of the ββα-Me-finger nuclease family typically hydrolyze both double-stranded (ds) and single-stranded (ss) DNA, as well as RNA at equal or similar rates [5], [20]. Members of this family contain a DNA/RNA non-specific endonuclease (NUC) domain consisting of a conserved Arg-Gly-His (RGH) motif [21], [22]. Side by side comparison of SID with the catalytic domain of SmNuc and the predicted domains of other family members is shown in Figure 1. As mentioned earlier, SID contains a perfectly conserved RGH motif with the critical histidine general base reside (*) and a conserved asparagine cation cofactor binding residue (**) along with the ββα-structural features. As can be seen in Figure 1, SID has more conserved amino acids in the catalytic domain than the mosquito *Culex quinquefasciatus* endonuclease (Cuqu Endo) that has been shown to be a catalytically active [10]. Sequence analysis of the *D. melanogaster* genome for open reading frames (ORFs) encoding proteins similar to SID revealed that there are seven putative nucleases with a high degree of similarity to SID with conserved RGH and cation binding residues (Figure S1A and B). In addition, several *Drosophila* species were found to have highly homologous *sid*-like genes including *D. sechellia* (95%), *D. simulans* (93%), *D. erecta* (82% and 72%), and *D. yakuba* (77%; data obtained from Flybase).

### The *sid* gene encodes a *bona fide* nuclease with cation dependence

**Figure 2:**
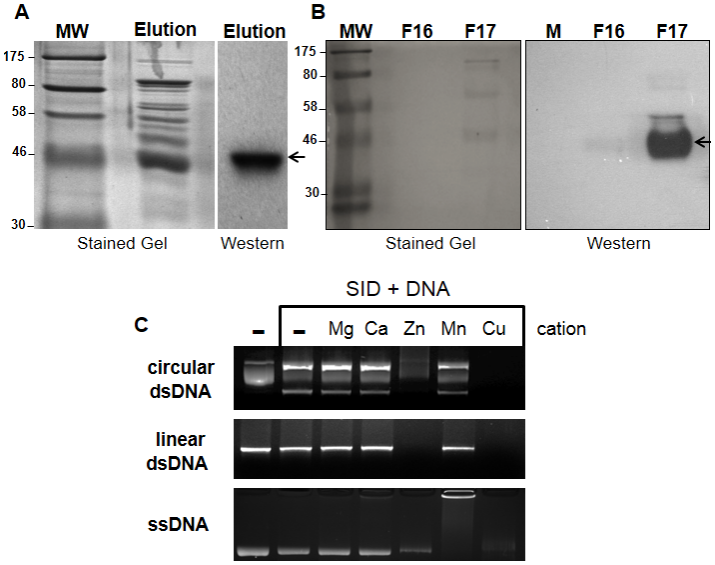
Expression, purification, and enzymatic activity of recombinant SID protein. (A) Histidine (6x) tagged SID protein expressed in bacteria was partially purified by immobilized nickel affinity chromatography. The partially purified fraction containing the SID protein (Elution) was subjected to SDS-PAGE electrophoresis and either stained *in situ* or transferred to a membrane (samples separated in duplicate) and hybridized with anti-Histidine antibodies. The recombinant SID protein was readily detected in the fraction containing 250 mM imidazole (see arrow). (B) Purification of the partially purified protein (from A) by size exclusion chromatography (Superose 12). After separation, the eluted SID protein was primarily detected in a single fraction (F 17; 1 ml) with anti-Histidine antibodies (arrow) as in part A. Molecular weight markers (MW; in kDa) are shown on the left side of gels. (C) Nuclease activity of partially purified (F 17) fraction on nucleic acid templates in the presence of common cations (1 mM). Circular plasmid double stranded (ds) DNA was degraded extensively in the presence of Cu^++^ and Zn^++^ but not when the enzyme was incubated along with other cations or in the absence of cation (–). The plasmid DNA contained supercoiled (middle band), nicked circular (top band) and linear DNA (bottom band). Degradation of linear DNA and single stranded DNA was also observed only in the presence of Cu^++^ and Zn^++^. All reactions were carried out for 2 hr at 37°C.

To evaluate the potential enzymatic activity of the recombinant SID protein, the ORF encoding the *sid* gene was cloned into a couple of bacterial expression vectors. Recombinant protein was first generated with a poly-Histidine tag and was partially purified by standard nickel column chromatography. Unfortunately, after repeated attempts, the eluted fractions containing the SID protein were found to be contaminated with several bacterial proteins (see Figure 2A). Fractions containing the enriched SID protein were therefore further purified by size exclusion chromatography to remove contaminating proteins (see Figure 2B). Western blot analysis with anti-Histidine antibodies revealed that SID was enriched in a single fraction (F17; black arrow in Figure 2B) with weaker reactivity in adjoining fractions. Since all members of the ββα-Me-finger nuclease family require cations for enzymatic activity [23], we incubated the fraction containing the SID protein (F17) with several common cations to determine if SID exhibited cation specificity for its activity. As shown in Figure 2C, when incubated with plasmid DNA and single stranded DNA, SID exhibited stronger nuclease activity in the presence of Cu^++^ and Zn^++^ with very weak activity with the other cations. Interestingly, when single stranded DNA was used, it was partially degraded with Mn^++^ and the majority remained complexed at the loading well (Figure 2C). Under such conditions, the recombinant protein may have stronger affinity for DNA generating large protein/DNA complexes that do not migrate into the gel. In addition, the enzyme was found to be fully active between a pH range of 5.0 and 7.5 and a salt concentration ranging from 0–200 mM (data not shown).

**Figure 3:**
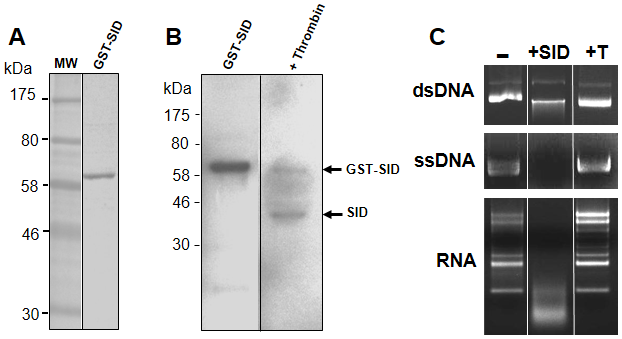
Expression and purification of GST-SID fusion protein. (A) A GST-SID fusion protein was expressed in bacteria and partially purified with glutathione beads. The GST-SID protein was separated as in Figure 1, and the eluted fraction was found to have few contaminants by protein staining (Coomassie R-250 blue). (B) Thrombin cleavage of the GST-SID fusion protein resulted in the release of the SID protein (arrow) as detected with anti-His antibodies (on the carboxyl-terminus of SID). (C) The thrombin-cleaved recombinant SID protein was examined for nuclease activity on DNA and RNA substrates in the presence of Cu^++^ (+SID) or in the presence of thrombin alone (+T). All reactions were carried as in Figure 1.

In order to further purify the SID protein, the *sid* gene was re-cloned downstream of the Glutathione-S Transferase (GST) gene along with the poly-Histidine tag. As can be seen in Figure 3A, the GST-SID fusion protein (∼68 kDa) was purified by affinity chromatography using a Glutathione-Sepharose matrix. After affinity purification, the recombinant SID protein preparation was found to contain very few contaminants as determined by protein staining (Figure 3A). Removal of the GST moiety from the His-tagged SID protein was accomplished through a thrombin cleavage site located between the GST and SID protein (Figure 3B). To determine the extent of protein cleavage, the thrombin treated samples were analyzed by western blot with anti-Histidine antibodies, which revealed that a significant proportion of the GST-fusion protein was cleaved. As expected from our previous results with partially purified SID, the thrombin treated protein was found to cleave plasmid DNA (dsDNA), single-stranded (ssDNA) and RNA when incubated in presence of Cu^++^ (+SID) and no activity was detected with thrombin alone (+T, Figure 3C). SID, which was partially purified by different affinity tags and purification schemes, was found to have similar nucleic acid cleavage activities and cation dependence as other members of the ββα-Me-finger nuclease family.

### Expression and localization of *sid* in the larval fat body and adult flies

**Figure 4:**
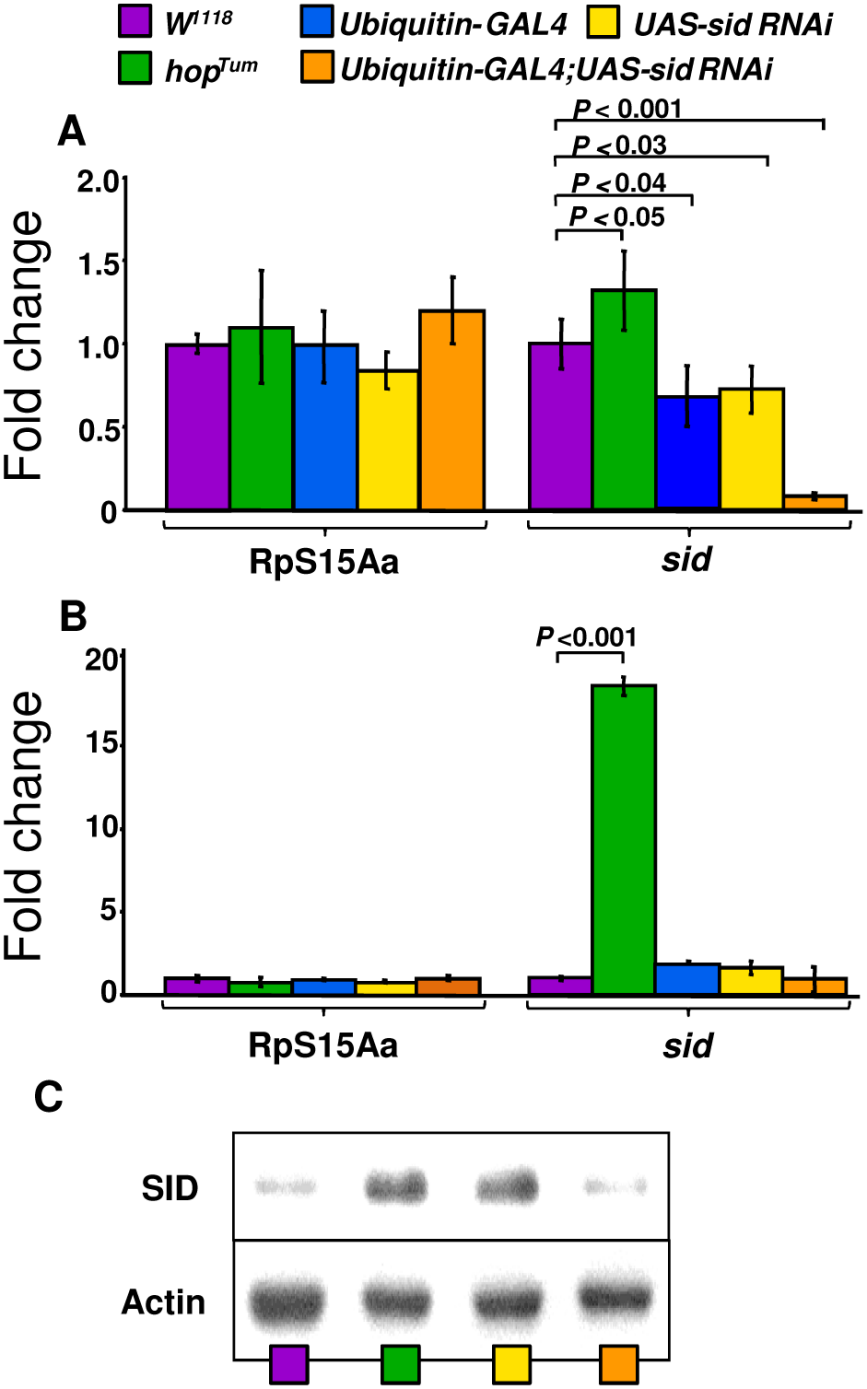
Expression profile of *sid* in the larval fat body and adult flies. (A) Expression of *sid* was analyzed by qRT*-*PCR in control flies *W^1118^* as well as the *hop^Tum^* mutant line and two transgenic fly lines, *UAS-sid RNAi* and *Ubiquitin-GAL4;UAS-sid RNAi*. Expression of *sid* in each sample was normalized to the expression of the ubiquitously expressed ribosomal RpS15Aa (CG2033) gene. Expression levels for both RpS15Aa and *sid* were calibrated to the wild type control levels, which were then assigned an arbitrary value of one, for each gene. Fold expression (numbers over bars) was determined by dividing the mean value derived from the *W^1118^* control flies by the mean value obtained from other fly lines (ie., *hop^Tum^ sid* value over *W^1118^ sid* value). Values were rounded up to the next decimal. Arrows represent fold up-regulation (up) or down-regulation (down). Each qRT-PCR experiment was performed in triplicate using RNA samples (cDNA) from the different genotypes. Bars represent the standard deviation from the mean. (C) Detection of SID protein by western blot analysis. Crude extracts from adult female flies were separated by SDS-PAGE, transferred to PVDF membranes and hybridized with rabbit anti- SID peptide antibodies as described in Materials and Methods.

*In situ* RNA hybridization with *sid* has revealed that this gene is primarily expressed in the fat body of developing pupae (data derived from the Berkeley Drosophila Genome Project; [24]). At the post pupae stages, the *sid* gene is primarily expressed in the larval fat body (FB). Although highly expressed in the adult FB, SID is also expressed in the head, midgut, tubule and spermatheca (virgin and mated; data from FlyAtlas; [25]). To confirm the expression of *sid* in larvae, fat bodies from third instar larvae of control (*W^1118^*) flies and four additional lines were examined by quantitative real time (qRT) PCR (Figure 4A). For this analysis, an additional fly line was included that was predicted to over-express the *sid* gene (*hop^Tum^*; [26]). The *hop^Tum^* transgenic fly line that expresses a constitutively active JAK (Janus) kinase, was previously shown to over-express the *sid* gene by ∼4 fold [26]. While expression of the *sid* gene was found to be slightly up-regulated (∼1.4 fold; Figure 4A) in *hop^Tum^* larvae, this gene was highly up-regulated (18.6 fold; Figure 4B) in adult flies. These results confirmed that *sid* is indeed up-regulated by JAK kinase over-expression in *hop^Tum^* flies. In addition, a *sid* RNAi line (*Ubiquitin-GAL4;UAS-sid RNAi)* was procured that should have reduced levels of *sid* gene expression. As expected, expression of *sid* was significantly down regulated ∼9 fold by expression of the *sid RNAi* construct in larval fat bodies (Figure 4A) but there was little if any effect in adult flies (Figure 4B).

### Analysis of levels of SID protein expression

The levels of SID (42 kDa) protein expression were examined in adult flies by western blots with anti-SID antibodies. Both *hop^Tum^* and *Ubiquitin-GAL4;UAS-sid* lines, which should over express the SID protein (see Discussion Section) were found to have similar levels of the SID protein of the predicted molecular weight of ∼42 kDa (Figure 4C). In contrast, protein extracts derived from wild type control (*W^1118^*) and *sid* RNAi (*Ubiquitin-GAL4;UAS-sid RNAi*) fly lines had significantly lower levels of the SID protein (Figure 4C). Since the wild type control and *sid* RNAi knockdown flies contained low levels of SID protein, it was not possible to precisely determine the fold differences between samples. As can be seen in Figure 4C, the *sid* RNAi line (*Ubiquitin-GAL4;UAS-dicer;UAS-sid RNAi*) had slightly lower levels of SID than wild-type fly line (*W^1118^*).

### Subcellular localization of SID *via* confocal microscopy

**Figure 5:**
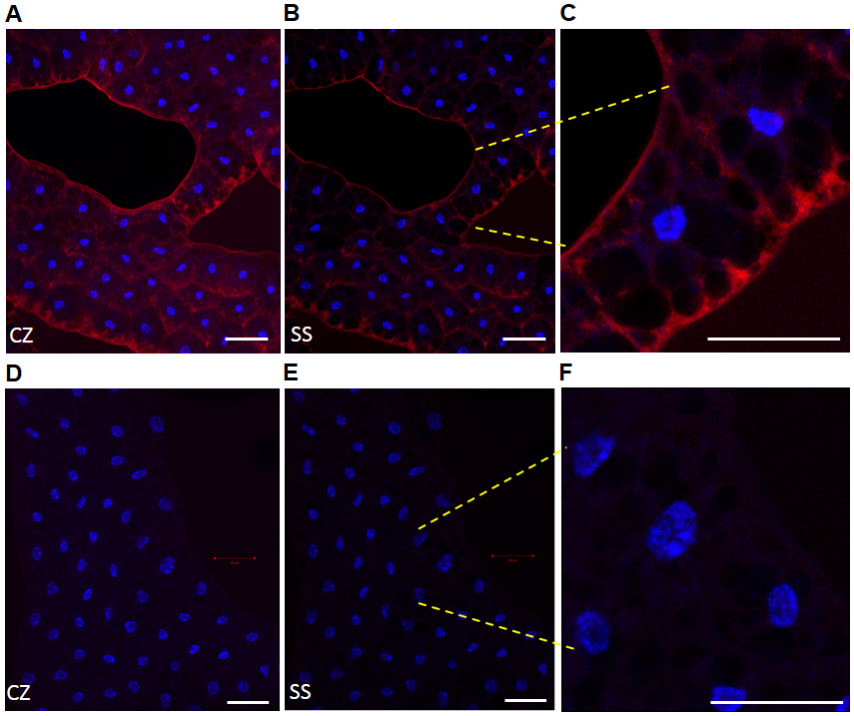
Expression of SID protein in the fat body of 3^rd^ instar larvae. FB optical sections from control flies were exposed to anti-SID peptide antibody (A–C) and the immune complexes detected with a secondary antibody (goat anti-rabbit) labeled with Alexa Fluor 568. Only FB slices that were incubated with both primary and secondary antibodies (A–C) resulted in Alexa staining (red). Controls FBs that were only incubated with secondary antibody did not show significant staining (D–F). DAP1 (1 µg/ml) was used to localize the nuclei of FB cells (blue). The captured Z-stack images were collapsed (CZ) to visualize the full intensity of the stained FBs (A and D) and compared to a single optical section (SS; B and E). As a reference point for single sections, the section (optical slice) with the brightest nuclear staining was used. White scale bars represent a 50 µm region and shown at the bottom right of each image. Note that images containing a few cells are shown at the right at a higher magnification (C and F; yellow dashes mark region) and all were adjusted to the same level of brightness (+20) to enhance the visualization of the image. Larvae from the control fly line *Ubiquitin-GAL4* were used in these studies. Images were captured under the same experimental conditions of micro-channel plate (MCP) gain and laser power output *via* a confocal fluorescence LSM 700 microscope.

In order to determine the cellular localization of the SID protein, anti-SID antibodies were generated and used to detect the SID protein by immunofluorescence microscopy in FB of third instar larvae. Analysis of FB from control flies revealed that the SID protein was primarily localized to the cytoplasm (Figures 5A–C). Note that the dark circular regions within the cells are lipid droplets of various sizes that are characteristic of FB cells (Figure 5C). These FB droplets are clearly traced with anti-SID antibodies indicating the presence of the protein in the cytoplasm. As expected, little if any staining was detected with secondary antibody alone (Figure 5 D–F). Interestingly, SID-derived peptides have been previously detected in cytoplasmic fractions of the hemocyte cell line Schneider 2 (S2) cell line by proteomic analysis (Peptide Atlas; [27]). An analysis of the deduced amino acid (aa) sequence of SID, predicted that SID lies outside of the membrane anchored *via* a transmembrane (TM) domain from aa 6–26 using a web-based TM prediction site (see Figure 5 legend TMHMM Server v. 2.0; http://www.cbs.dtu.dk/services/TMHMM/). As shown in Figure 5, the immunofluorescence results show strong staining to the membrane regions as well as the cytoplasm in normal flies; thus indicating that SID is both cytoplasmic and associated with FB cellular membranes.

### *Sid* gene expression is affected by bacterial infection and oxidative stress

**Figure 6:**
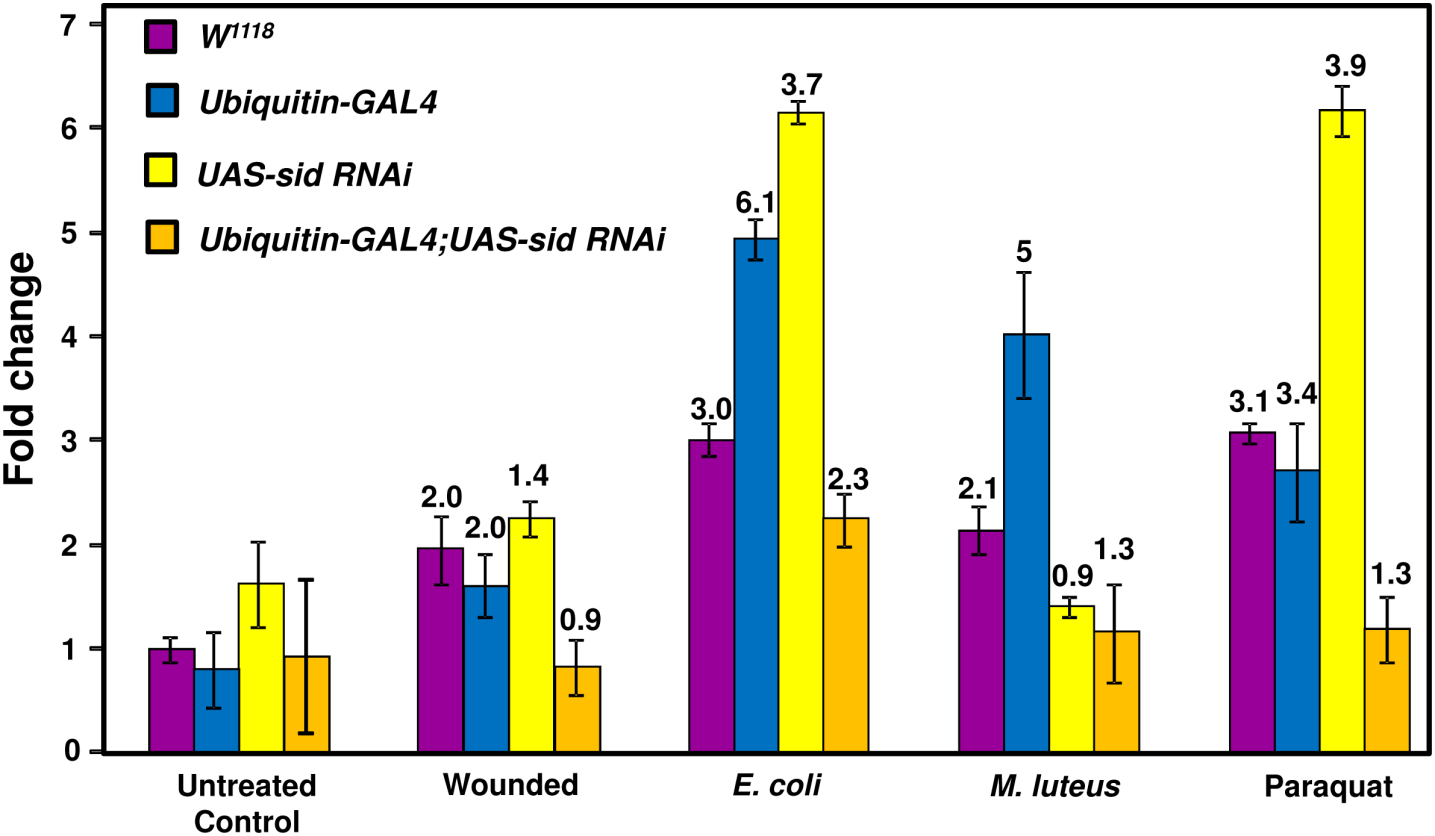
Analysis of *sid* expression levels in untreated and infected flies. Expression of *sid* was analyzed by qRT-PCR in control (not infected) and flies infected with *E. coli* flies or *M. luteu*s bacteria. *sid* expression levels were calibrated to the uninfected *w^1118^* control level, which was assigned an arbitrary value of one. Fold expression (numbers over bars) was determined by dividing the mean value derived from the infected flies by the mean value obtained from uninfected flies (for each line; i.e., *W^1118^* infected over uninfected *W^1118^*). RpS15Aa expression values were very similar across all lines and not used to adjust overall expression levels.

As mentioned earlier, the *sid* gene was found to be induced by 5.2 fold after 1.5 h of infection with a mixture of *M. luteus* and *E. coli* bacteria and its expression remained elevated up to 48 h post infection [4]. Although *sid* expression was detected *via* microarray analysis, its expression was not verified by other means. For this reason, *sid* expression was analyzed after infection of flies with either *M. luteus* or *E. coli* bacteria to determine if either type of bacteria specifically induced expression of this gene. For this analysis, RNA samples were prepared from flies infected with either *E. coli* or *M. luteus* bacteria after 48 h and *sid* expression levels were compared by qRT-PCR (see Materials and Methods for details). As shown in Figure 6, infection of control (*W^1118^*) flies lead to an increase of *sid* gene expression of ∼3 and ∼2 fold when infected with *E. coli* and *M. luteus* bacteria, respectively. Interestingly, the highest up-regulation (5 to 6 fold) was noticed in the control *Ubiquitin-GAL4* fly line when infected with either bacteria that was not noticed in the other fly lines. Flies that expressed the *sid* RNAi construct (*Ubiquitin-GAL4;UAS-sid RNAi*) exhibited lower *sid* up-regulation when infected with *E. coli* (∼2 fold) but not when infected with *M. luteus* bacteria. These findings confirm the previously reported microarray data that a mixture of *E. coli/M. luteus* bacterial infection was able to induce *sid* expression. Our infection analyses revealed that *E. coli* and *M. luteus* bacteria elicited similar effects on *sid* expression that was not statistically significant (Figure 6). The effects of aseptic puncture was also analyzed and wounding was found to have a less than 2-fold effect on *sid* expression on all fly lines analyzed after 48 hrs post puncture (Figure 6). As mentioned earlier, a genome wide expression analyses of genes activated by oxidative stress (paraquat ingestion), revealed that *sid* gene expression was up-regulated by ∼3 fold [4]. Due to these results, the effects of oxidative stress on *sid* gene expression were analyzed by qRT-PCR to confirm its up-regulation after paraquat treatment (see Figure 6). For this analysis, total RNA was obtained from two separate groups of females placed in media containing 10 mM paraquat or normal media. As can be seen in Figure 6, after 48 h of exposure to paraquat, *sid* expression was induced in the control lines by ∼3 fold but no induction was detected in the *sid* knockdown fly line. Taken together, these results confirm the induction of *sid* expression after bacterial infection and exposure to paraquat-induced oxidative stress.

### Effects of wounding on fly viability and quantification of bacterial loads post-infection

**Figure 7:**
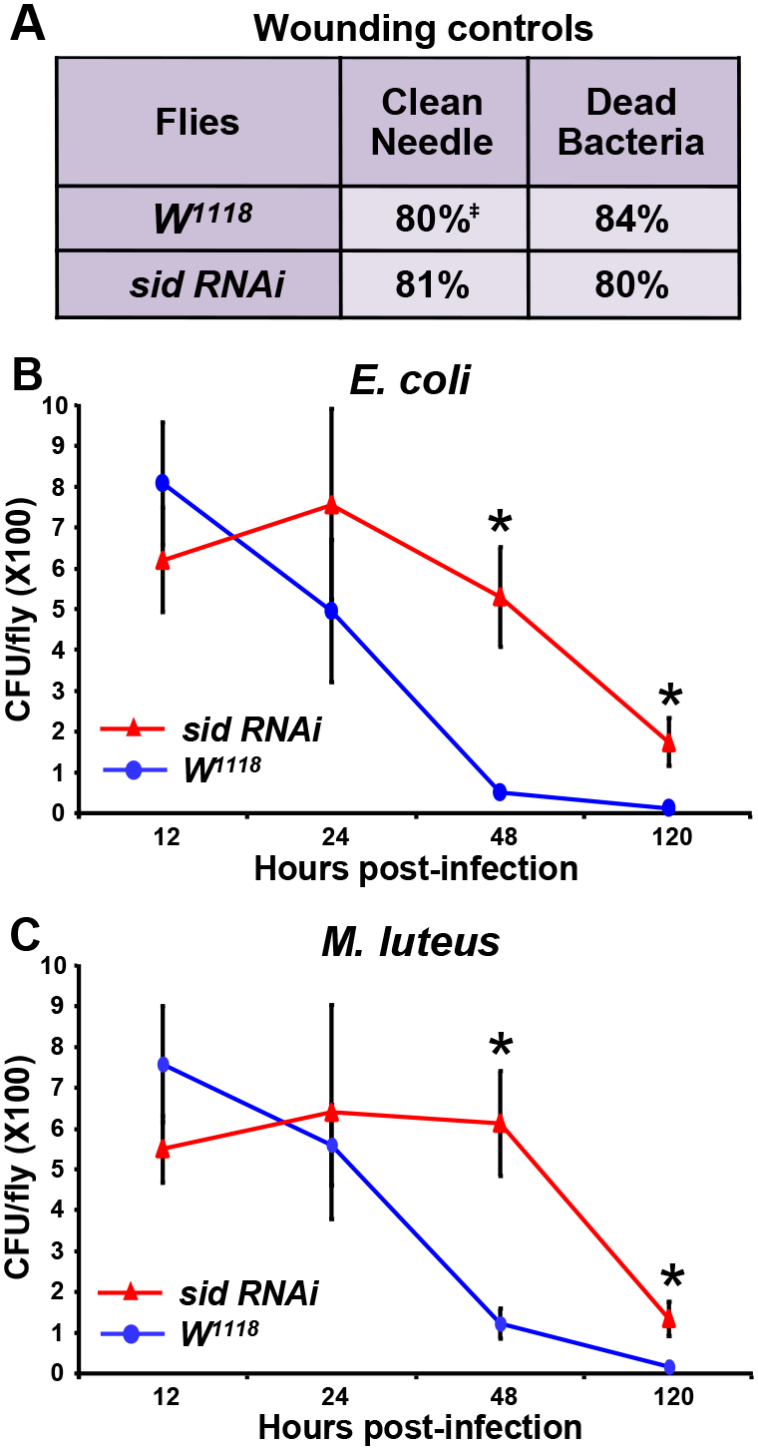
Analysis of survival after wounding and infection. (A) The survival rate (**^‡^**) of flies after wounding with a clean needle or when injected with dead bacteria was analyzed after 7 days. The number of colony forming units (CFU) was also analyzed to determine the bacterial load over time in *W^1118^* (blue line) and *Ubiquitin-GAL4;UAS-sid RNAi* (red line) flies infected with *E. coli* (B) or *M. luteus* (C). Significant differences in bacterial load between the two fly lines were determined *via* two-tailed paired Student’s *t*-tests with a *P* value of <0.01 (*).

Two additional wounding controls were performed in which flies were either punctured using a clean dry tungsten needle or flies were punctured with a suspension of dead bacterial cultures. In both instances, wounded control (*W^1118^)* and *sid* knockdown (*Ubiquitin-GAL4:UAS-sid RNAi*) flies, exhibited a similar ∼80% survival rate of after 7 days of incubation (Figure 7A). Infection of control flies (*W^1118^*) and *sid* RNAi (*Ubiquitin-GAL4:UAS-sid RNAi*) did not result in a significant difference in the total number of colony forming units (CFU) after 12 or 24 hr of infection with either bacteria (Figures 7A/B). While the CFU counts dropped by ∼4 to 10 fold in control flies after 48 hrs of infection, the CFUs remained relatively constant (at 12–24 hr infection levels) in the *sid* knockdown flies (Figures 7A/B). However by 5 days (120 hrs) of infection, the CFU counts dropped significantly and to similar levels with either bacterial strain. These results indicate that the infected flies were eventually capable of reducing bacterial loads in control and *sid* knockdown flies.

### Bacterial infection reduces viability in *sid* deficient flies

**Figure 8:**
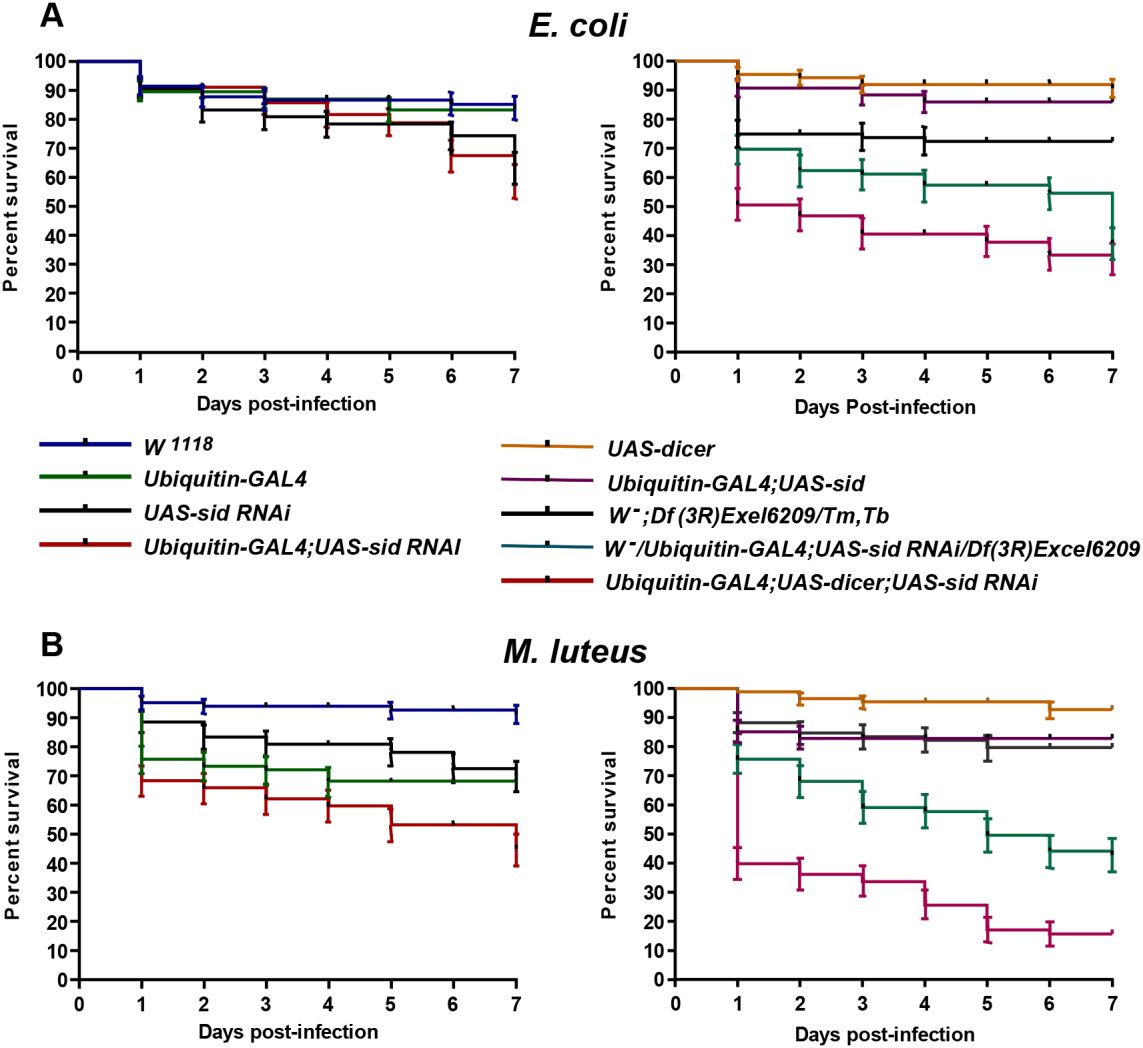
Down-regulation of *sid* gene expression resulted in reduction of fly viability after infection with bacteria. Twenty five 2–3 day-old female animals were infected with *E. coli* (A) or *M. luteus* (B), from each indicated fly genotypes. All vials containing the post-infection experimental and control groups were maintained at 29°C on regular fly medium for seven days. Every day the total number of live and dead flies was recorded. Each time point represents the average of three independent trials. *W^1118^* wild-type *c*ontrol flies were compared with experimental fly lines (*P*<0.0001, Log-rank test for trend).

Since *sid* expression is increased after *E. coli* bacterial infection, an attempt was made to determine if SID plays a role in protection against infection. To address this possibility, an infection study was carried out with flies that either over-express *sid* or are deficient in *sid* expression. As can be seen in Figure 8A, infection with *E. coli* bacteria of control (*W^1118^*) or the *sid* over-expressing transgenic fly line (*Ubiquitin-GAL4;UAS-sid*) did not have much of an effect on the viability of infected flies during a 7 day time course. However, infection of *sid-*deficient flies that express the *sid* RNAi construct (*Ubiquitin-GAL4;UAS-dicer;UAS-sid RNAi*) resulted in a significant ∼50% and ∼70% reduction of viability when infected with *E. coli* or *M. luteus* bacteria, respectively as early as 24 h post infection (Figure 8B and D). In these *sid*-RNAi expressing flies, the reduction of viability was more pronounced when infected with *M. luteus* bacteria reaching ≥80% after 5 days of infection (Figure 8D). Reduction of viability after infection with either type of bacteria of the *Ubiquitin-GAL4;UAS-sid RNAi* fly line reached a maximum of ∼60% and co-expression of the *UAS*-*dicer* construct further reduced the viability of *sid* RNAi flies to greater than 75% by seven days post infection (Figures 8B and D). Comparison of the effects of infection on a deficiency *Df(3R)Exe;6209/Tm,Tb* line carrying a deletion of *sid* on one chromosome to a derivative that expresses the *sid*-RNAi construct (*W^−/^Ubiquitin-GAL4;Df(3R)Exel6209/UAS-sid RNAi)* revealed that this *sid*-RNAi/deficiency line was consistently more susceptible to infection by either bacteria than the deficiency alone or the RNAi expressing lines (Figure 8C and D). These results indicate that lines with lower *sid* expression are more susceptible to infection with either *E. coli* or *M. luteus*. Interestingly, ubiquitous over-expression of *sid (Ubiquint-GAL4; UAS sid)* did not confer higher resistance to infection with either *E. coli* or *M. luteus* as the effects of infection of this transgenic fly line was similar to those of controls (Figure 8 C and D).

### Effects of paraquat-induced oxidative stress on fly viability

**Figure 9:**
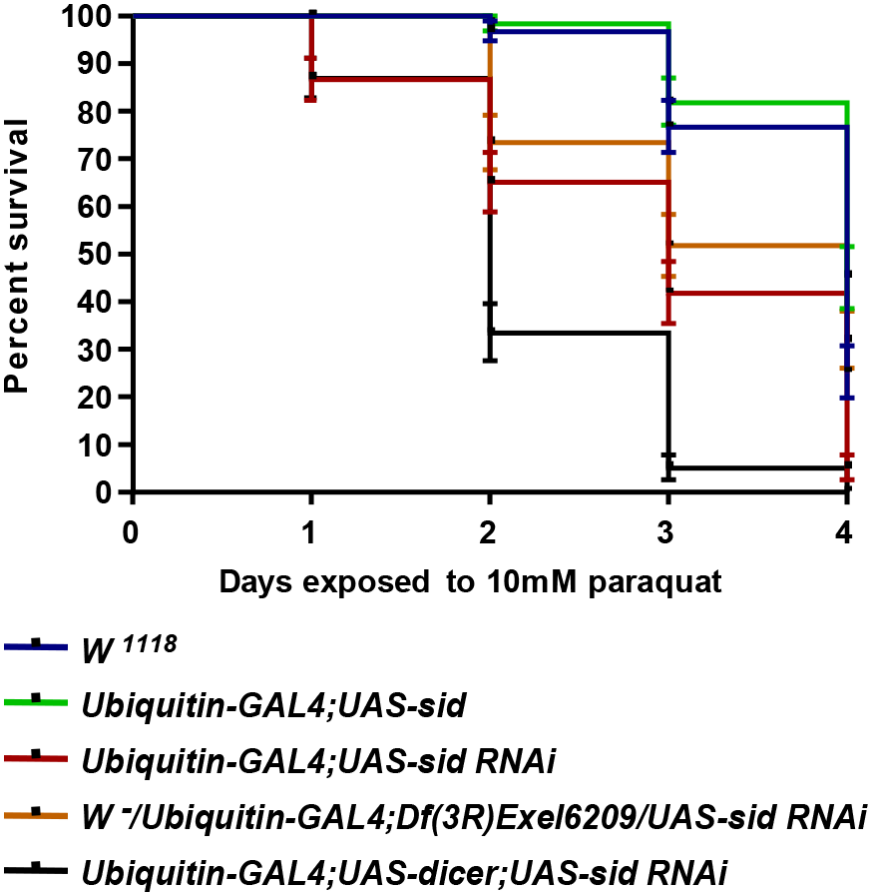
Fruit flies deficient in *sid* expression are more susceptible to paraquat-induced oxidative stress. In a typical viability experiment, twenty 2–3 day-old female flies were placed in a vial with solid medium containing 5 mM and 10 mM paraquat and maintained at room temperature for four days. The fly lines used in this study are shown on the right side of the graph. Every day the total number of live and dead flies was tallied. Three replica vials were used for each fly strain with the same indicated genotypes. Each bar represents the standard error of the mean of three independent experiments. *W^1118^* wild-type *c*ontrol flies were compared with experimental fly lines (*P*<0.0001, Log-rank test for trend).

To determine the effect of *sid* down regulation on fly survival after exposure to oxidative stress, several fly lines were exposed to various concentrations of paraquat. In preliminary tests of the effects of paraquat on fly survival, all of the *sid* deficient flies exposed to 15 mM paraquat exhibited almost complete loss of viability by 48 h of exposure; and therefore the effects on gene expression and long-term viability could not be determined (data not shown). Due to the high toxicity of paraquat at the 15 mM concentration, a lower concentration of 10 mM was used in all subsequent experiments. As can be seen in Figure 9, after 2 days of exposure to 10 mM of paraquat approximately ∼70% of *sid*-RNAi flies (*Ubiquitin-GAL4*;*UAS-dicer;UAS-sid RNAi)* died while the viability of control flies (*W^1118^* and *Ubiquitin-GAL4;UAS-sid*) was not significantly affected (>90% survival). Furthermore, *sid* deficient (*W^−^/Ubiquitin-GAL4;Df(3R)Exel6209/UAS-sid RNAi*) flies were more susceptible to paraquat exposure after 2, 3, and 4 days, as compared with *Ubiquitin*-*GAL4; UAS-sid RNAi*. In contrast, the *sid* over expressing line (*Ubiquitin-GAL4;UAS-sid*) line exhibited higher viability when exposed to 10 mM paraquat as compared to all other fly lines. A can be seen in Figure 9, a loss viability of ∼90% was observed by three days of exposure to 10 mM paraquat in *sid* knockdown flies (*Ubiquitin-GAL4;UAS-dicer;UAS-sid RNAi*) while only a loss of ≥30% of viability was seen in the control lines. The negative effects of oxidative stress on fly survival was significantly exacerbated by four days of exposure with a total loss of viability in the *Ubiquitin-GAL4*;*UAS-dicer;UAS-sid RNAi* and the deficiency line (*W^−^/UbiquitiGAL4;Df(3R)Exel6209/UAS-sid RNAi)* while the control lines retained greater than 20% viability. These results confirm the sensitivity of the *sid*-deficient lines to exposure to paraquat and the induction of oxidative stress.

## Discussion

The only insect ββα-Me-finger nuclease that has been well characterized is Cuqu Endo from the mosquito *Culex quinquefasciatus* [10]. This salivary enzyme apparently assists in blood meal intake by lowering the viscosity of ingested DNA or by acting as an anticoagulant by producing small DNA haptamers [10]. SID shares a highly conserved catalytic domain with CuQu Endo and was therefore predicted to exhibit nuclease activity (Figure 1). As expected, recombinant SID was found to have similar catalytic properties as other nuclease family members with the ability to cleave both DNA and RNA when incubated with specific cations (Figures 2 and 3).

During bacterial infection, fruit flies mount a vigorous immune response consisting of activation of professional phagocytes, a strong melanization response, and the release of several distinct AMPs [28]–[32]. The AMP response is mainly responsible for the destruction of invading organisms and results in the generation of a large amount of bacterial debris that is efficiently removed by professional phagocytes. During this response, a large array of secreted peptidases, proteases and other enzymes are also involved in the dissolution of debris generated by pathogen destruction. Some of the major bacterial components released during this process are nucleic acids and this material needs to be removed efficiently. Our prior work has demonstrated that reduction of DNase II activity severely reduces fly viability after bacterial infection [1]. Since DNase II is primarily used to degrade DNA within phagocytes, we hypothesized that other nucleases would be required to remove excess nucleic acids released in the hemolymph and interstitial fluid.

Septic injury results in the activation of hundreds of genes that are regulated by well characterized signaling pathways that include Toll, Imd, JAK-STAT and JNK [28]–[30]. Another pathway that is involved in the response to septic injury is the MAP kinase pathway [18]. This pathway regulates a small subset of genes and flies carrying a mutation in the *Mekk1* gene are highly susceptible to paraquat-mediated oxidative stress [18]. A genome wide expression analysis of wild type and *Mekk 1* mutant flies revealed that fifteen genes were repressed while five others were induced after infection of mutant flies [18]. Of the fifteen genes that were repressed, two of them were genes encoding the nucleases *dnase II* and *sid*. The authors suggested that the repressed genes are involved in the response to infection and oxidative stress and also concluded that *Mekk1* is required in adult flies for oxidative stress protection [18]. Interestingly, induction of *sid* by bacterial infection was completely abrogated in *Mekk1* mutant flies thus implicating the *MAP* kinase pathway in the induction of this gene [18].

Over-expression of the GATAe protein in the larval FB results in activation of *sid* implying that these transcription factors regulate expression of *sid* and that GATA binding sites are present within its promoter region [33]. For this reason, an *in silico* analysis of the upstream region of *sid* was performed that revealed two perfect GATA motifs within its proximal promoter region. In addition to the GATA binding sites found in the *sid* promoter region, one near perfect Dorsal/Rel binding motif (GGGATTTtCt; lower case not a match) was also localized near the two GATA binding sites in an arrangement similar to that seen in other AMP gene promoters [33]. The GATA transcription factor, Serpent, is a key regulator of many well-known immune response genes that collaborates with the Rel-transcription factors, Dorsal, Dif and Relish [33], [34]. As mentioned earlier, *sid* is over-expressed in *hop^Tum^* constitutive JAK kinase overexpressing line by ∼4 fold [26] and our expression analysis confirmed this observation (see Figure 4). Due to these results, we analyzed the *sid* promoter region and found a perfect Stat92E binding consensus motif (^−239^ TTCnnnGAA^−231^ [35]). Based on its previous detection in several microarray analyses, it appears that *sid* is a *bona fide* member of the AMP response that can be activated by more than one signal transduction pathway. Future experiments which are beyond the scope of this work, will address the transcriptional and signal transduction pathways that regulate *sid* gene expression by distinct stress inducers.

Over-expression of the *sid* gene was originally detected by infection of flies with *M. luteus* [36] and also by co-infection of flies with *E. coli* and *M. luteus* bacteria [2] . We determined that this response was similar in intensity when flies were infected with either *E. coli* or *M. luteus* bacteria (Figure 6). However, *M. luteus* bacterial infection of *sid* RNAi flies resulted in a ∼70% loss of viability, while *E. coli* bacteria infection only reduced viability by ∼50% after one day of infection (Figure 8). It is therefore tempting to speculate that in normal and *sid*-overexpressing lines, infection by *E. coli* bacteria induced higher levels of *sid* that resulted in a protective effect against infection.

Exposure of *D. melanogaster* to the Gram negative bacteria *Erwinia carotovora carotovora* 15 (ECC15) leads to productive but non-lethal infection and a potent induction of the immune response [37]. A global expression analysis of infection with ECC15 bacteria revealed that *sid* was not induced even after 16 h of infection [37]. However, infection with the highly pathogenic *P. entomophila* resulted in the induction of a large number of genes including *sid*, which was up-regulated by 9.9 and 46.9 fold after 4 and 16 h of exposure, respectively [3]. Interestingly, exposure of flies to a non-pathogenic variant of *P. entomophila* did not induce *sid* expression [3]. Infection with pathogenic *P. entomophila* resulted in irreversible damage to gut cells *via* the generation of reactive oxygen species that resulted in inhibition of protein translation and impairment of local immune and cellular repair responses [3]. In a recent genome wide analysis of genes of genes induced by the Toll and Imd pathways, *sid* was found to be activated in uninfected Toll-gain of function mutant (*Tl10b*) flies by 3.2 fold [38]. In the same study, *sid* was found to be induced by bacterial infection and in flies impaired in Imd signaling (*relish* mutant) but this induction was not detected in flies that do not activate the Toll pathway (*spaetzle* mutant [38]). Taken together, this data would predict that *sid* expression would only be induced by gram positive bacteria (Toll-dependent) and not by gram negative bacterial infection; but this was not what we observed. As mentioned earlier, *sid* is upregulated in the JAK kinase constitutive (*hop^Tum^*) overexpressing line and therefore *sid* expression is not totally dependent on activation of the Toll pathway [26].

Although our results indicate that wounding did not have a significant effect on fly viability on *sid* deficient flies as compared to controls (Figure 7A), two previous reports have revealed that *sid* was activated by 9.3 fold by media injection alone after 6 h [39] and 4.6 fold after 2 hours of wounding [40]. In the latter study, injection of trypsin resulted in a 7.2 fold increase in *sid* expression as compared to 4.6 fold by puncture alone after 120 min [40]. Interestingly, *sid* induction by injection with trypsin occurred as early as 30 minutes (2.48 fold) as compared to puncture alone (1.3 fold; M. Juarez and W. McGinnis personal communication). In our study, aseptic puncture with sterile needles embedded in LB media did not cause a significant increase in *sid* expression, as compared with unwounded flies, when analyzed after 48 h post-puncture (Figure 6). Altogether, previous reports and our findings indicate that the increase of *sid* expression after wounding is a rapid event (within 30 minutes) that decreases to near baseline levels after 48 h. The rapid induction of *sid* expression indicates that its induction is a response to stimuli that mimics activation of the serine proteolytic pathway activated during the fly’s response to wounding [40].

It has been well documented that resistance and tolerance are important factors to consider when analyzing the effects of pathogens on infected hosts [39], [41]–[45]. Resistance and tolerance are context-dependent and can be measured by various parameters that include pathogen load and health of the host (see review by Ayres and Schneider, 2012). For example, it has been demonstrated that a single gene mutation in the melanization pathway of *Drosophila* can affect both resistance and tolerance to a variety of pathogens [39]. Our experiments have revealed that SID partially protects the host against pathogens and may indeed be involved in both resistance and tolerance to bacterial infections.

Accumulation of DNA due to the absence or mutation of DNases has been linked to pathological conditions in various organisms due to increase inflammation and the over-production of cytokines and other effectors [1], [46]–[55]. As can be seen in Figure 7B and C, reduction of *sid* expression resulted in higher bacterial loads by 48 h post-infection with either *E. coli* or *M. luteus* infection as compared to wild-type control flies. It is important to point out that the most significant drop in fly viability in *sid*-deficient flies occurred within the first 48 h of infection and this correlates with the lack of pathogen clearance during this period (Figure 8B and D). Although the *sid*-deficient flies eventually clear the pathogens, they appear to be slower at achieving clearance perhaps due to excess pathogen-derived nucleic acids in the system (Figure 7B and C). These results point to lower resistance of the *sid*-deficient flies to bacterial infection as compared to control flies. However bacterial load was subsequently reduced to near control levels by five days of infection implying that other anti-microbial mechanisms are involved in pathogen clearance. If the main function of SID during infection is to clear excess nucleic acids generated by bacterial lysis by the normal AMP response, then reduction of this enzyme would result in excess nucleic acids in the infected flies. Excess nucleic acids could result in an impairment of the immune response that fails to reduce bacterial load after 48 h of infection (Figure 7B and C). However reduction of bacterial loads by 5 days post-infection does not explain why *sid* knockdown flies continue to die past this time point. This would imply that SID reduces tolerance to infection possibly due to increased levels of circulating nucleic acids that reduce host viability resulting from the generation of a toxic factor(s) due to an altered immune response. Excess DNA in flies has been shown to lower resistance to bacterial infection possibly due to excessive signaling by nucleic acid receptors such as EYA [1], [56]. Our results indicate that the response to infection in *sid*-deficient flies is biphasic with lower resistance to infection at earlier time points and reduced tolerance after reduction of bacterial loads at the longer time points most likely due to the effects of nucleic acid accumulation.

In other work, *sid* was found to be up-regulated by ∼6 fold by parasitic wasp invasion [57] and was one of a few genes whose expression increased in flies selected to resist parasitic invasion [58]. A comparison of strategies used by different wasp parasitioids revealed that a strain that induces a strong immune response, *Leptopilina boulardi*, also induced *sid* expression by 7.2 fold [59]. However, no induction was seen with a strain (*L. heterotoma*) that does not induce a vigorous immune response. As shown in Figure 6, *sid* was also induced by ∼3 fold after exposure to oxidative stress and this induction appears to enhance fly viability as *sid* knockdown flies were severely affected by exposure to paraquat resulting in a total loss of viability by four days of exposure (Figure 9). In conclusion, the induction of SID by bacteria, parasitic wasps, and oxidative stress indicates that this protein is an important protective component of the AMP response.

## Supporting Information

Figure S1 **Peptide sequence of the CG9989 (SID) protein and comparison with other potential fly nuclease family members.** (A) The complete peptide sequence of the CG9989 open reading frame is shown with the critical RGH catalytic residues and the region used to generate anti-SID antibodies are shown in bold and underlined. (B) Alignment of SID with putative *Drosophila melanogaster* nuclease family members. Sequence alignment was performed with ClustalW (http://www.ebi.ac.uk/Tools/msa/clustalw2/). Shading was performed with Boxshade (http://www.ch.embnet.org/software/BOX_form.html). The protein with the highest identity scores are shown in the order of similarity to SID (e-values on the side). The putative proteins encoded by the CG33346 ORF, CG12917, and CG14062 were found to have a high degree of similarity to SID (see e values next to alignment). Four additional ORFs were found with lower overall similarity but a perfectly conserved RGH motif (red shaded residues) and cofactor binding residue (see red shade) and these are CG14120 (7.8 e-7), CG3819 (4.5 e-6), CG14118 (4.6 e-6), and CG6839 (3.4 e-4). Dashes at the end of the alignment indicate that the protein sequence continues beyond that point. Note that the CG14120 ORF encodes a very large polypeptide that contains two nearly identical nuclease domains that have much lower similarity with the SID protein but both have a conserved RGH motif.
(PDF)
