# Supplementary material for: Cloning and Characterization of a Novel Drosophila Stress Induced DNase
Source: PLoS One. 2014 Aug 1;9(8):e103564. doi: 10.1371/journal.pone.0103564 (PMC4118900; doi:10.1371/journal.pone.0103564)
Supplement: Figure S1 — Peptide sequence of the CG9989 (SID) protein and comparison with other potential fly nuclease family members. (A) The complete peptide sequence of the CG9989 open reading frame is shown with the critical RGH catalytic residues and the region used to generate anti-SID antibodies are shown in bold and underlined. (B) Alignment of SID with putative Drosophila melanogaster nuclease family members. Sequence alignment was performed with ClustalW (http://www.ebi.ac.uk/Tools/msa/clustalw2/). Shading was performed with Boxshade (http://www.ch.embnet.org/software/BOX_form.html). The protein with the highest identity scores are shown in the order of similarity to SID (e-values on the side). The putative proteins encoded by the CG33346 ORF, CG12917, and CG14062 were found to have a high degree of similarity to SID (see e values next to alignment). Four additional ORFs were found with lower overall similarity but a perfectly conserved RGH motif (red shaded residues) and cofactor binding residue (see red shade) and these are CG14120 (7.8 e-7), CG3819 (4.5 e-6), CG14118 (4.6 e-6), and CG6839 (3.4 e-4). Dashes at the end of the alignment indicate that the protein sequence continues beyond that point. Note that the CG14120 ORF encodes a very large polypeptide that contains two nearly identical nuclease domains that have much lower similarity with the SID protein but both have a conserved RGH motif. (PDF) [file pone.0103564.s001.pdf]

A)

M P D L K Y M L **T I L S L Y F F V G S V Q A N C** L I D L A H L N A
N Y V Y L S Q N N G V Y D I Q R S D I V E I H Q T L Y L L C N G G
L H R T T F L C R Y D S V F S P A L S S A A C A P P D P V V V K V
P D T S C S I P S A T F A V G F S F N G R F M E L Y R N C F D G Y
S L A F Q H S I Y K A Y R Y V N T V P R P N P T W Q S D Q L S G G
F D N A Y E G R A T Q A C L L T N L G A V Q P Q C K F D **R G H** M T
P A S A F I S T E L K K S T F R Y L N A I P Q Y R G V N R G K W K
A V E T W V N N M V R G L Y D N P I I N N V Q I P R T Y D V L K V
C I G A L G V H R L R H N T N N N M I P I Y L L D N N K I P V P E
W M Y K I V S H L S G D K W V M L T Y N D V S L P N Q Q A L N Q I
C H V I P C H P G L N L N T K D V G H T V C C D P Y R F I T I N A
P H L T G V C

B)

```
Name                                                                          E value
CG9989(SID)1 --------------------------------------------MPDLKYMLTILSLYFFV 0
CG12917    1 -----------------------------------MKIAPMDCSYITLFIYIYFICFLAWE 2.3 e-26
CG14062    1 ---------------------------------------------MLRILLLLLAFCAL 1.9 e-22
CG14120    1 ----------MWSNRCSIFILALLVASGAVKVSGRLPRILPESLAEGDEGVTVRGDCEFK 7.8 e-7
CG3819     1 ----MKCLVLIAFSGLFLAS---------AQARILPAEDLPWELPEVPVAVNEIEPRAAG 5.7 e-6
CG14118    1 MMLQFIGLILAVVILREIRAREAIATIPDVGQIIKDLDGLHIDGEMFHQGSPCRVDVQND 5.9 e-6
CG6839     1 ----MKCIRFSLLVVGLLAAPAAWARVPCPEVELPPVVEDDGIFERIAVAPPQPVGRAGA 4.4 e-4


CG9989    18 GSVQANCLIDLAHLNANYVYLSQNNGVYDIQRSDIVEIHQTLYLLCN-----GGLHRT--
CG12917   27 VHGDCQILQYMVEQSNGIFTYRDASGSIQLQRLETVPSGVTLLVYCS-----PSVFKETV
CG14062   15 AHGQCRFTRAQVQGTNRIFMVRGQNRQLSLKRTASSAVGETLQMWCN-----PRDIVATT
CG14120   51 VNGDLNDPAPLFSRHNSY---EIIVPDPTDTVRLVNGELLDMFCPGVGFAAPFVNRWQVT
CG3819    48 CSIKIRSSELKDPQPLLIKSDTSEIVGFSDTGYVDVDKDKTIEFHCTSSLASPLSGKSVT
CG14118   61 LPRLDKVQPLYLRPGTDLYWLPNAYGHLEVQRGASIELHCSHSFAPANGESLDAKLRSIR
CG6839    57 CSVTIRGG-LPSPEPVYLKTDSEDFYPFSDVGVMEFESGGSLQLWCPSGFNTHSENLLTT


CG9989    72 FLCRYDSVFSPALSSAACAPPDP------VVVKVPDTSCSIPS-----ATFAVGFSFNG-
CG33346    1 -----------------------------------------------MYLVGFKYGN- 1.2 e-42
CG12917   82 CQDN--GQFSVPLPMRCLSPMQP------VTKHIRDGDCAGN--------LYAVGYTIDG-
CG14062   70 CQAGRVPAFQPPLPMTCRAAPAA------ITTPVQDRRCPAT--------MYRVGYNVGNN
CG14120  108 ATCLQNKYFLVDDLIYPFANFSC------TAWPIFTALRSGKDCNGGTDLVQVGFEVEDGG
CG3819   108 AKCVGGTTFKIDDKEHDLSAIKC------TSWPVFVGKKSGSSCNGGTTLIKVGFELSGS
CG14118  121 VKCVQDTTFEWMGAKIHFSDFVCNHSMPYTVERLDRSCGSDTPSPSTSYLYRVGYDTGDG
CG6839   115 ASCVSGTTFSVGGSNFEFKDLYC------KSWPGFKAVKSGATCNGG-IVIRVGFEITSS


CG9989   120 RFMELYRNCFDGYSLAFQHSIYKAYRYVNTVPR--PNPTWQS---------DQLSGGFDN
CG33346   11 TFMELYRSCYDARTMKAYFSINTVYPTNLKSDR--PPTVFDKDGIITPA--DEATFQMNS
CG12917  126 KDLELYRTCFDCGQGRLVYSQSDVYYKTFFPKR--PFVEFVADEMFSPQ--EAAAYMKSN
CG14062  117 QFLELYRACFDTRAVRAIFVEHRVYGKPFYITR--PCVQFSSDGVISGA--DEASYTVRN
CG14120  163 -FLQSYELCHDAEAEATRYVHHVLYPSSYDYQHGVARPNFLELDFYGGRDVNTKYTQVQQ
CG3819   162 RFATQYEVCFNEDEEVTRYVYHRLEPGNNYYATGVDRITFGAGGYFAGKNVDKLYTQAVQ
CG14118  181 RFVATMELCHDPNQLRTHYAHHQLTPANVHFQKKLKRPRFSTAGHFTG-------FDMAR
CG6839   168 RFAEQMQICFNEEEEVTRYTRHKLEPGSNYYETGVARITFQTAGFFDGKNVDKLYTQATQ


CG9989   169 AYEGRATQACLLTNLGAVQPQCKFDRGHMTPASAFISTELKKSTFRYLNAIPQYRGVNRG
CG33346   67 IYNRFEYLFGSGQTYVPSSRSLSFDRGHLTPVADYSFPKILRQTNKYLNVVPQYYNINRS
CG12917  182 IYFAFKCIYGDDQSYLQNANYLVINRGHMVASADFLFTDQMGSTFRYLNVVPQFKSINDG
CG14062  173 IHGTFRRLFGNNQNYIPNNRDVIINRGHLAASADFFFGDQLCATFKYVNA PQFKSINDG
```

```
CG14120   222 NITISNILGLDASPYFNFSDDRILSRGHMIAKTDQIFGAAQHTTFLFINVAPQWQTFNGG
CG3819    222 KETIDKELDMDSSRFFDSAKNIFLARGHMGAKADFVFAPEQRATFLFINAAPQWQTFNAG
CG14118   234 IYSPKSQEKLMVPGLIDVKSGLFLARGHLTAKADLIYASQQKSSFNYMNVAPQWQSFNGG
CG6839    228 LETINNELGGDAEKYFDSSSNVYLARGHLGAKADFDYAPEQRATFLFINAAPQWQTFNAG

CG9989    229 KWKAVETWVNNMVRGLYDNPIINNVQIPRTYDVLKVCIGALGVHRLRHNTNNNMIPIYLL
CG33346   127 NWKIVENWVR--------------GQK----DVLNVCTGALGVLDLLNRSQKSVSIYLAP
CG12917   242 NWEKIERWVR--------------SQIPK-SSYFRVKSGGIGILTLPDTRGFLQSAFLAG
CG14062   233 NWETIERFVR--------------NSVTG-NNFVNVRTGARGVLSLPSGN-RPKNVFLSG
CG14120   282 NWEKVETSVR--------------KFVADRNLTTDCYTGTWGVSTLPDVDGIERELYLDF
CG3819    282 NWARVEDGVR--------------AWVAKENKHVECWTGVWGV TLPNKNGEQRQLYLSH
CG14118   294 QWSKLEESTR--------------QYVARSGITATVYTGIYG----EMKVAGSKVLHMTT
CG6839    288 NWARVEDGLR--------------AWVSKNKLNVNCYTGVYGVTTLPNKDGVETPLYLAV

CG9989    289 DNNK---IPVPEWMYKIVSHL-SGD-KWVMLTYND--VSLPNQQALNQICHVIPCHPGLN
CG33346   169 NKN-----PVPRWTYKIIRSL-TTGIKYVILTSNN--GWETQQPNPASVCKVTACPRTLN
CG12917   287 SKI-----PVPEWTYKAVRDA-TGNGLYVFLTYNS--TFQMEKPPCLAICYPVNCPIHLP
CG14062   277 NRN-----PVPQWMYKIVRNA-NNQPIVAFLTLNN--IYARQRPAAPNFCQPVNCPVALV
CG14120   328 DENNNGLIPVPKIYFRVVIDRVTRE-GIVLIGINNPYLTLEQIQKDYILCQDIGHQLSWL
CG3819    328 DNNGNGLIPVPKLYFRVVIEP-STKKGIVLIGVNNPHLSLEEIKRDYILCTDVSDRINWI
CG14118   336 NANNIGVVAVPQLFYRVLIDEGHPTRGIALVGVNNPHATLAQIHESYIICDPVEESVQWL
CG6839    334 DDNNNGLIPVPKLYFRVVIDP-SSHRGIVFVGVNNPHLTEEQIKRDYVICDDVSDQVTYI

CG9989    342 --------LNTKDVGHTVCCDPYRFITINAPHLTGVC-------
CG33346   221 ---------PTG-TGYTFCCDPIDFIRRNVPNLAGVCWDNTENV
CG12917   339 ---------NNPNDGYTFCCDPKRFPY-----------------
CG14062   329 ---------NTAQAGFSFCCNPATFRP-----------------
CG14120a  387 TWY-----KEDLHEGYSYACSVEDFIEVVKDLPLEDLHTNGILG....
CG3819    387 SWK-----KTDITAGYSYACEVPEFRKKVTHLPEFSVSGLLV--
CG14118   396 SWLHKSNAKGNLKNGYLYACSVANLARAVGHLPRPLLEVDELLT
CG6839    393 NWK-----TTDIKAGWSYACEVADFLKTVKHLPALTAKGGLLV-
```
